# Clinical Outcomes of In Vitro Fertilization (IVF) Versus Intrauterine Insemination (IUI) in Infertile Patients: Toward Evidence-Based Fertility Planning

**DOI:** 10.7759/cureus.92511

**Published:** 2025-09-17

**Authors:** Tanzeela Bano, Maryam Ali Shaheen, Nosheena A Shabbir, Aisha Khan Jadoon, Samreen Ameen, Maryam Atta, Saiqa Noor, Muhammad Iftikhar Khattak, Amna S

**Affiliations:** 1 Department of Obstetrics and Gynecology, Corniche Hospital, Abu Dhabi, ARE; 2 Department of Obstetrics and Gynecology, Sheikh Khalifa Bin Zayed Al Nahyan Hospital, Muzaffarabad, PAK; 3 Department of Obstetrics and Gynecology, Aga Khan University Hospital, Karachi, PAK; 4 Department of Surgery, Azad Jammu and Kashmir Medical College, Muzaffarabad, PAK; 5 Department of Medicine, Azad Jammu and Kashmir Medical College, Muzaffarabad, PAK; 6 Department of Gynecology, District Headquarters Hospital (DHQ) Timergara, Lower Dir, PAK; 7 Department of Research and Development, Health Services Academy, Islamabad, PAK; 8 Department of Obstetrics and Gynecology, Hamdard University, Karachi, PAK

**Keywords:** amh, chi-square, infertility, iui, ivf, pakistan, pcos, reproductive success, treatment outcomes

## Abstract

Background

Infertility poses a significant reproductive health burden, and assisted reproductive technologies (ART) such as intrauterine insemination (IUI) and in vitro fertilization (IVF) are widely used treatments. This study aimed to compare the clinical outcomes of IVF and IUI in infertile women to inform evidence-based fertility planning.

Methods

A total of 300 infertile women were analyzed, evenly distributed into IVF (150, 50%) and IUI (150, 50%) groups. Baseline demographics, hormonal markers, and infertility characteristics were assessed. Clinical outcomes, including pregnancy and live birth rates, were compared. Statistical analyses included t-tests, chi-squared tests, correlation analysis, logistic regression, and machine learning modeling.

Results

The overall clinical pregnancy rate was 119 (39.7%). IVF yielded a significantly higher pregnancy rate (69, 46%) compared to IUI (50, 33.3%) (χ²=4.15; p=0.042). Similarly, live birth was more frequent in the IVF group (61, 40.7%) than the IUI group (41, 27.3%) (χ²=4.84; p=0.028). Adverse outcomes included multiple pregnancies in 15.9% of IVF vs. 6% of IUI cases. Higher success was noted in women aged <30 (IVF: 52.3%; IUI: 39.2%). Anti-Müllerian hormone (AMH) and follicle-stimulating hormone (FSH) were significantly correlated with pregnancy outcomes (AMH: r=0.38 and p<0.001; FSH: r=-0.25 and p=0.011). Logistic regression identified IVF (OR=1.68), AMH (OR=1.52), and FSH (OR=0.81) as significant predictors. A random forest model achieved 73.1% accuracy.

Conclusions

IVF demonstrated superior clinical outcomes over IUI. Hormonal profiling and early IVF consideration may improve ART success in selected infertile patients.

## Introduction

Infertility is a significant and growing health concern affecting approximately 15% of couples worldwide, as reported by the World Health Organization (WHO) [1,2]. Defined as the inability to conceive after one year of regular, unprotected intercourse, infertility can be caused by a variety of factors affecting both males and females. Female infertility can stem from such things as ovulatory disorders, endometriosis, polycystic ovary syndrome (PCOS), or blocked fallopian tubes, while male infertility is usually due to problems with sperm count, motility, or morphology [3,4]. At times, both partners have a role to play in infertility, and of course, there is also unexplained infertility. In addition to the biological issues, infertility can also be emotionally, socially, and financially distressing. Emotionally, couples will often go through a grief process, feeling frustrated and depressed and experiencing a toll on relationships and their well-being [5]. Socially, the stigma of infertility can lead to feelings of isolation, especially for women experiencing infertility. Financially, treatment options (i.e., in vitro fertilization (IVF) and intrauterine insemination (IUI)) are expensive, and IVF in particular can be in the upper ranges of money spent [6,7]. According to the American Society for Reproductive Medicine (ASRM), a single cycle of IVF in the United States ranges between $12,000 and $15,000, plus expenses that include medication, embryo freezing, and/or genetic screening [8]. The financial burden can dissuade couples from seeking fertility options or lead to multiple cycles without producing success. 

The growing prevalence of infertility has contributed to the use of assisted reproductive technologies (ART) because ART is vital for couples trying to achieve a pregnancy. ART consists of various medical techniques and procedures that manipulate the eggs and sperm outside of the human body in order to aid with conception [9]. In fact, the most common ART methods are IVF and IUI. IVF involves retrieving the eggs from the ovaries and using sperm from a male partner or sperm donor to fertilize the eggs in a laboratory outside the body. The fertilized eggs are grown into embryos for a few days in the laboratory and can be inserted back into a woman's uterus. IVF can be an excellent option for someone with a severe infertility problem like blocked fallopian tubes or males experiencing male factor infertility [10]. With IVF, the success rates (for people with the same cause of infertility) are typically quite high, but not uniform across the process, and vary widely depending on many different factors such as age, infertility factor, and the types of eggs and sperm. If someone is under the age of 35, the success rates of IVF are generally 40-50% per cycle, but this rate typically decreases as the age increases [3,5]. IVF is a success and can be quite effective; however, it is intrusive both emotionally and financially, and it can be expensive. Importantly, every decision with IVF and ART processes needs to consider a risk-benefit analysis of invasive treatment, where some of the complications could include ovarian hyperstimulation syndrome (OHSS) or complications with multiple pregnancies [11].

IUI is a less invasive and less costly procedure involving the direct insertion of sperm into the uterus. The procedure will be performed during ovulation. Women who suffer from unexplained infertility or mild male factor infertility and/or have ovulatory concerns will be the most suitable candidates [12]. Success rates of IUI are lower than IVF at approximately 5-20% per cycle depending on the patient and the reason for infertility [7]. Though IUI is a less invasive and less costly treatment than IVF, you may need to undergo multiple cycles before achieving a successful pregnancy [13]. According to the Centers for Disease Control and Prevention (CDC), approximately 7.3 million women in the United States seek fertility treatment. IUI is more than likely the first step onto your journey of conception after a diagnosis and prior to initiating IVF [14]. As evidenced, the decision between IVF and IUI largely considers the cause of infertility, the age of the patient, as well as the financial and emotional implications. While infertility is in itself an arduous process, there is a full spectrum of decisions to make within infertility [15].

While IVF and IUI are becoming increasingly common, there remain multiple gaps in the literature with regard to the comparative effectiveness of these treatment modalities across different patient populations. Most available literature has focused on reporting the success rates of each procedure separately. However, there have been no comprehensive retrospective studies that have compared IVF and IUI outcomes, complications, and patient experience. The objective of this study is to address these gaps to take place in a retrospective analysis of IVF and IUI outcomes across a variety of patient populations [16]. Specifically, the purpose of this study is to compare success rates, complications, and patient outcomes of IVF and IUI by focusing on how patient characteristics, such as age, infertility diagnosis, and lifestyle factors, can affect treatment outcomes [17].

The broad research questions this study aims to answer are as follows: How does the overall success rate of IVF compare to that of IUI, and how does the success rate differ among patients by group: male infertility, female infertility, and unexplained infertility? What complications are associated with IUI or IVF? How do patient characteristics, including age, diagnosis, and individual lifestyle, contribute to success with IVF and IUI? In answering these questions, we will present a more thorough and nuanced understanding and examination of the real-world utility of these two treatments. The results will be useful for clinicians and patients in making more informed decisions when considering fertility treatment options, which aim to optimize patient care and ultimately enhance treatment success. Additionally, our findings will contribute to awareness within the field of reproductive medicine while providing a basis for future reproductive research on ART outcomes among patients in IUI and IVF treatment.

## Materials and methods

Study design

This research used a retrospective observational cohort study design to compare two treatments for primary infertility: IVF and IUI. The data for this study consisted of clinical data from existing fertility centers in Pakistan. A retrospective observational cohort design was employed to understand how IVF and IUI perform as treatments in real life and to find predictive associations across patient and treatment variables. By working with historical data, the study analyzed treatment success patterns in the hope of contributing to evidence-based fertility care.

Study setting and population

The study population initially consisted of 700 patients (men and women), aged between 20 and 45 years, who had undergone either IVF or IUI. Participation was contingent upon the patient's medical file containing complete records (clinical, diagnostic, treatment, and outcome data). However, the analysis was conducted on a selected subgroup of 300 infertile women, with 150 patients in each group (IVF and IUI). These 300 patients were chosen from the larger 700-patient dataset based on the availability of comprehensive medical records. Patients who were undergoing alternative experimental procedures (as per the waiver from PubMed Central (PMC)) or who had incomplete files were excluded from the analysis to maintain the credibility and consistency of the data. The selected subgroup appears to be diverse in terms of age, causes of infertility, and socioeconomic status and is representative of many fertility patients nationally in Pakistan.

Data collection

The data was obtained from structured medical records, aggregated into a dataset with 700 rows and 50 variables. The data provided a wide range of patient characteristics including demographic factors (age, gender, body mass index (BMI), educational level, and socioeconomic status) and clinical factors including diagnosis of the infertility (e.g., PCOS, male factor, tubal obstruction), type of infertility (primary or secondary), comorbidities (e.g., hypertension, diabetes, obesity), and reproductive history. Diagnostic and laboratory factors consisted of hormone profile data (follicle-stimulating hormone (FSH), anti-Müllerian hormone (AMH), and thyroid function tests) and semen analysis factors. Treatment factors consisted of the number of IVF/IUI cycles, various medications (medication types and dosage; Clomid, letrozole, FSH) used, details regarding embryo transfer, and embryo quality scores. Documented outcomes included clinical pregnancy, live birth, miscarriage, ectopic pregnancy, and patient satisfaction scores. Lastly, behavioral factors, such as smoking status, alcohol consumption, and mental health (e.g., anxiety and depression), were included in the data.

Exploratory data analysis (EDA)

The first step in the analysis was to conduct EDA to get a sense of how the data were distributed across variables and the overall structure of the dataset. For continuous variables, descriptive statistics including means, medians, and standard deviations were computed, while frequencies or percentages were computed for categorical variables. Data visualization techniques, including histograms, boxplots, and scatter plots, were used to identify any trends, outliers, or distributional assumptions. Patterns of missing data were determined, and actions were taken to ameliorate or address missing data with either suitable imputation or case deletion to remove the potential impact of missing data. EDA also facilitated the identification of key variables that contributed to treatment success and guided the selection of predictors for modeling.

Statistical analysis

The statistical analysis was performed using IBM SPSS Statistics for Windows, Version 26.0 (Released 2019; IBM Corp., Armonk, New York, United States). Categorical variables were analyzed using chi-squared tests to examine associations between treatment outcomes and factors such as infertility diagnosis and medication use. Independent-samples t-tests were used to compare the means of continuous variables (e.g., age, hormone levels) between groups with different treatment outcomes. One-way analysis of variance (ANOVA) was applied to demonstrate the differences in multiple educational or socioeconomic groups regarding variables, including AMH. Pearson's correlation coefficients were calculated to examine the possible relationships of age with AMH or FSH. All statistical tests were two-tailed, and a p-value of less than 0.05 was considered statistically significant.

Predictive modeling

In addition to inferential statistics, predictive modeling was conducted to identify the most important predictors of treatment success. The primary outcome variable for modeling was binary (treatment success: Yes/No). Logistic regression was applied to estimate the odds of successful IVF or IUI based on independent predictors such as age, BMI, AMH, PCOS status, medication type, and behavioral factors like smoking and alcohol use. To further enhance prediction and variable importance ranking, a random forest classifier was employed. This machine learning model captured nonlinear interactions and provided insights into the relative contribution of each predictor. Model performance was evaluated using accuracy, sensitivity, specificity, and area under the receiver operating characteristic (ROC) curve. A fivefold cross-validation approach was adopted to ensure the robustness and generalizability of the results.

Ethical considerations

This analysis was conducted on anonymized data without direct patient identifiers. Ethical principles were followed in line with the Declaration of Helsinki, and all identifiable patient information was removed prior to analysis. The study adhered to standards for the ethical use of retrospective data and was approved by Abbas Institute of Medical Sciences (approval number: 1855/AIMS/2024).

## Results

Demographic characteristics

**Figure 1:**
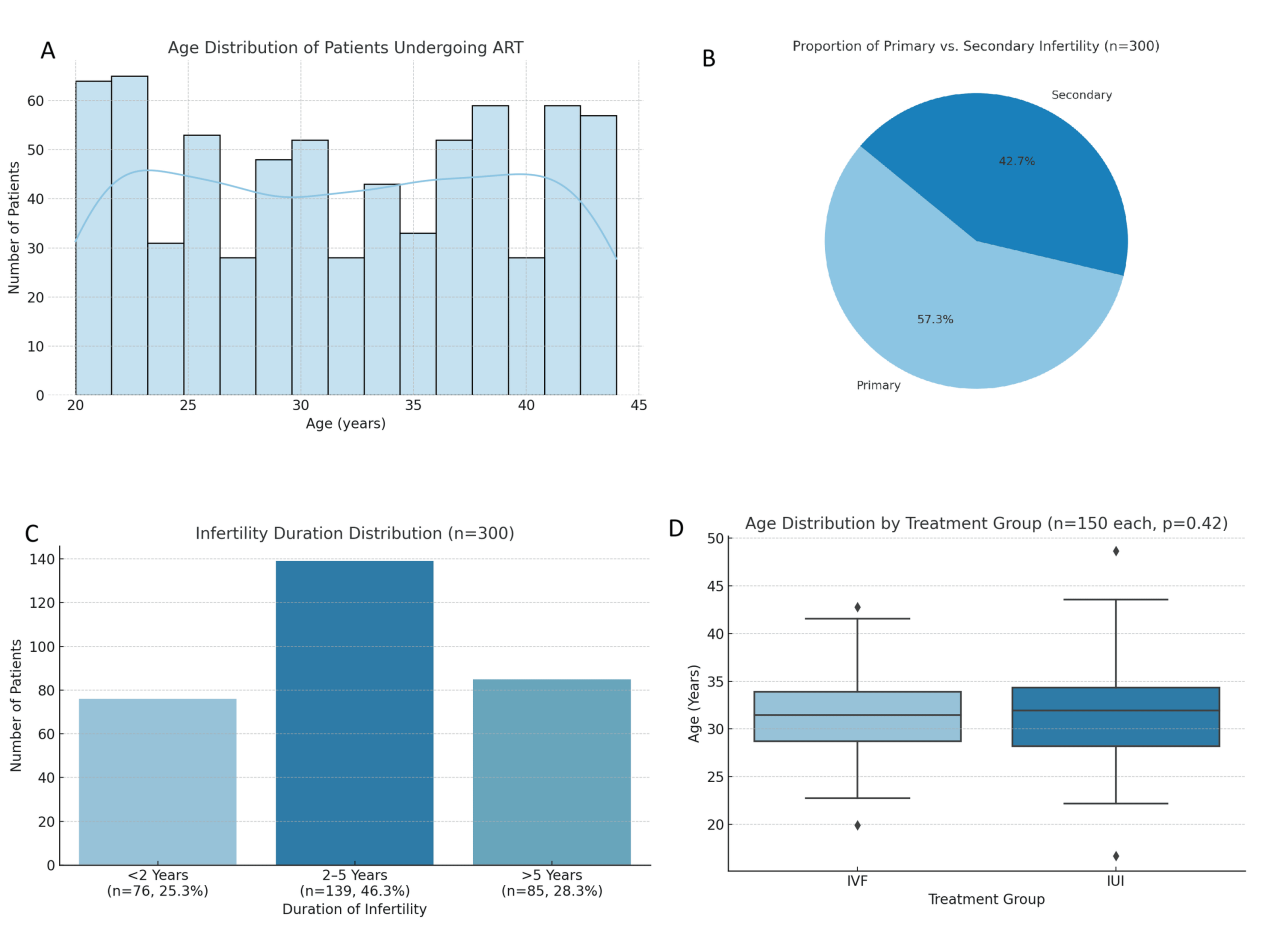
Demographic characteristics of patients undergoing ART. (A) A histogram displaying the age distribution of all patients (n=300), showing a relatively uniform spread between ages 21 and 44 years, with a mean of 31.5 years. The distribution is shown separately for patients receiving IVF and IUI to allow comparison between treatment groups. (B) A pie chart illustrating the proportion of primary infertility (57.3%) versus secondary infertility (42.7%) among patients. (C) A bar chart representing the duration of infertility: 25.3% of patients experienced infertility for less than 2 years, 46.3% for 2-5 years, and 28.3% for more than 5 years. (D) A boxplot comparing the age distribution by treatment group (IVF vs. IUI), showing no statistically significant difference between groups (p=0.42), confirming demographic comparability ART: assisted reproductive technology; IUI: intrauterine insemination; IVF: in vitro fertilization

The study included 300 infertile female patients undergoing ART, with equal representation from two treatment modalities, namely, IUI and IVF, comprising 150 patients (50%) in each group, as shown in Table 1. Male patients were not included in the analysis, as the study specifically focused on female infertility outcomes. The average age of participants was 31.5 years, with a standard deviation of 4.5 years, ranging from 21 to 42 years (as shown in Table 1), and a median age of 32 years. Information on parity or marital status was not included in the dataset and was therefore excluded from the analysis (Figure 1).

**Table 1:** Baseline demographic characteristics of the study participants (N=300) IUI: intrauterine insemination; IVF: in vitro fertilization

Characteristic	IUI group (n=150)	IVF group (n=150)	Total (N=300)
Age (years)			
Mean±SD	31.6±4.5	31.4±4.6	31.5±4.5
Range	21-42	21-42	21-42
Median	32	32	32
Type of infertility			
Primary	86 (57.3%)	86 (57.3%)	172 (57.3%)
Secondary	64 (42.7%)	64 (42.7%)	128 (42.7%)
Duration of infertility			
<2 years	38 (25.3%)	38 (25.3%)	76 (25.3%)
2-5 years	70 (46.7%)	69 (46%)	139 (46.3%)
5 years	42 (28%)	43 (28.7%)	85 (28.3%)
Mean duration±SD	3.9±1.8	3.9±1.8	3.9±1.8

Out of the 300 patients, 172 (57.3%) were diagnosed with primary infertility, and the remaining 128 (42.7%) with secondary infertility. Regarding infertility duration, 76 women (25.3%) had experienced infertility for less than two years, 139 (46.3%) for 2-5 years, and 85 (28.3%) for more than five years. The mean duration of infertility across all patients was 3.9 years with a standard deviation of 1.8 years. These statistics indicate that most women had been trying to conceive for 2-5 years at the time of treatment. The treatment distribution was evenly divided between the two groups. A total of 150 patients (50%) underwent IUI, and 150 patients (50%) received IVF. The equal distribution provided a balanced basis for the comparative evaluation of clinical outcomes between the two treatment types.

Independent-samples t-tests and Mann-Whitney U tests were conducted to assess the baseline equivalence between the IUI and IVF groups. There were no statistically significant differences in age (p=0.42) or duration of infertility (p=0.38) between the two treatment groups. Hormonal variables, including AMH, FSH, luteinizing hormone (LH), and estradiol (E2), were also not significantly different at baseline (all p>0.05), confirming that the two groups were comparable and suitable for outcome comparison.

EDA

**Figure 2:**
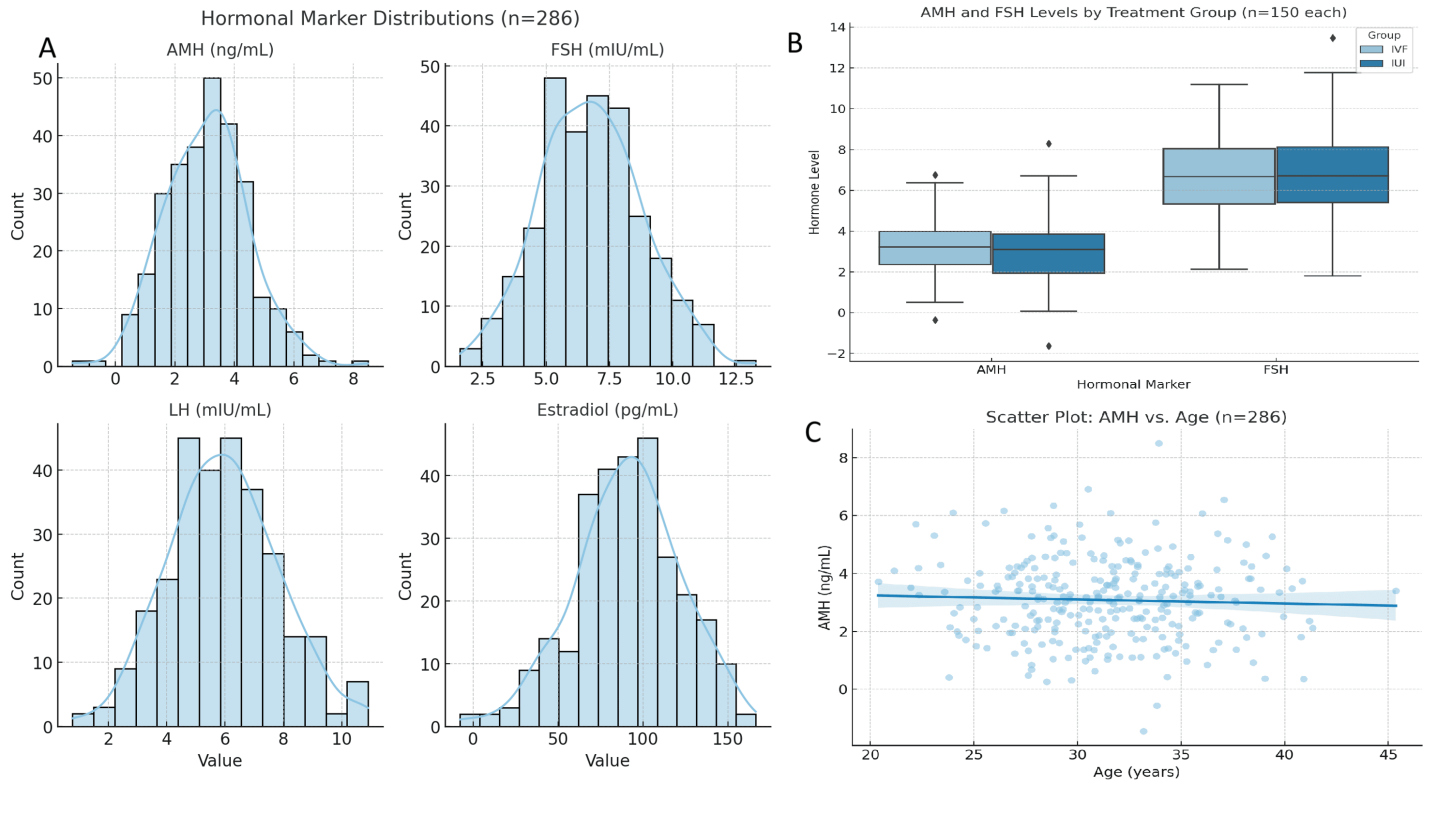
Hormonal marker distributions and comparative analysis by treatment group. (A) Histograms showing the distributions of key hormonal markers across the cohort (n=286): AMH, FSH, LH, and E2. All values fall within clinically expected ranges for women undergoing ART. (B) Boxplots comparing the AMH and FSH levels between IVF and IUI treatment groups (n=150 each). IVF patients exhibited slightly higher AMH and lower FSH levels, though differences were not statistically significant (all p>0.05). (C) Scatter plot displaying the relationship between AMH levels and patient age. A weak but negative correlation was observed (r=-0.19), consistent with known age-related decline in ovarian reserve AMH: anti-Müllerian hormone; FSH: follicle-stimulating hormone; LH: luteinizing hormone; E2: estradiol; ART: assisted reproductive technology; IUI: intrauterine insemination; IVF: in vitro fertilization

Among the 300 patient records, 286 (95.3%) were fully complete, while 14 records (4.7%) had missing data, particularly in hormonal variables (Table 2). These incomplete entries were excluded from the analysis using listwise deletion, ensuring data reliability without introducing imputation bias. The mean AMH level was 3.1 ng/mL (SD=1.4), mean FSH was 6.8 mIU/mL (SD=2.1), LH averaged 5.9 mIU/mL (SD=1.9), and mean E2 level was 85.3 pg/mL (SD=32.1). These values fall within the expected clinical ranges for patients undergoing ART. Frequency analysis showed that exactly 150 patients were treated in each group (IUI and IVF), 172 patients had primary infertility, and 128 had secondary infertility. Additionally, 119 (39.7%) of the patients achieved clinical pregnancy, while 181 (60.3%) did not. Normality was assessed using the Shapiro-Wilk test. Age and FSH were normally distributed (p>0.05), while AMH and E2 deviated from normality (p<0.05), indicating the need for non-parametric testing where appropriate (Figure 2). No extreme outliers were observed using ±3 standard deviation cutoffs from the mean. Comparing the IUI and IVF groups, there were no statistically significant differences in key demographic and clinical variables. IVF patients had a slightly higher average AMH level (3.3 ng/mL) compared to the IUI group (2.9 ng/mL) and marginally lower FSH levels (6.6 vs. 7 mIU/mL). However, these differences were not statistically significant. Age, LH, and E2 levels were also similar between the groups.

**Table 2:** Exploratory data analysis of the study variables IUI: intrauterine insemination; IVF: in vitro fertilization; AMH: anti-Müllerian hormone; E2: estradiol; FSH: follicle-stimulating hormone; LH: luteinizing hormone

Variable	IUI group (n=150)	IVF group (n=150)	Total (N=300)	P-value	Notes
Data completeness	-	-	286 complete (95.3%); 14 missing (4.7%)	-	Missing mainly in hormonal variables; listwise deletion used
AMH (ng/mL)	2.9±1.3	3.3±1.5	3.1±1.4	0.09	AMH and E2 non-normal (Shapiro-Wilk (p<0.05))
FSH (mIU/mL)	7.0±2.2	6.6±2.0	6.8±2.1	0.13	Normally distributed
LH (mIU/mL)	-	-	5.9±1.9	0.11	-
E2 (pg/mL)	-	-	85.3±32.1	0.08	Non-normal distribution
Primary infertility (n, %)	86 (57.3)	86 (57.3)	172 (57.3)	-	-
Secondary infertility (n, %)	64 (42.7)	64 (42.7)	128 (42.7)	-	-
Clinical pregnancy (n, %)	50 (33.3)	69 (46)	119 (39.7)	0.042	-
No clinical pregnancy (n, %)	100 (66.7)	81 (54)	181 (60.3)	-	-

Treatment outcomes

**Figure 3:**
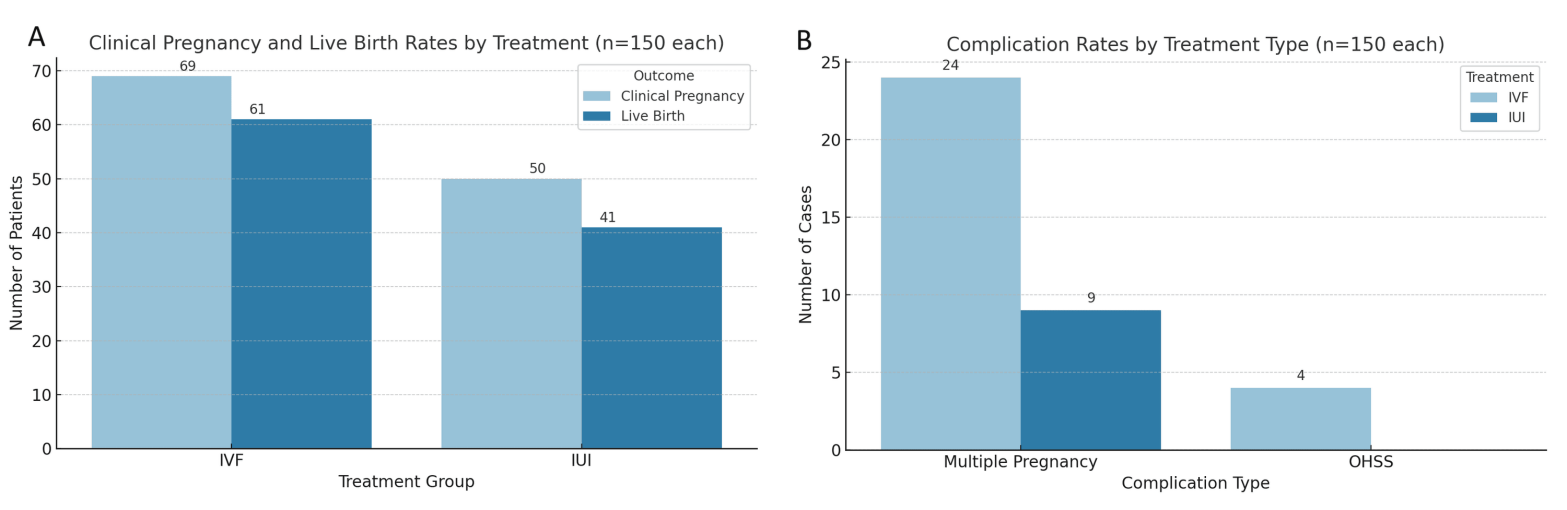
Treatment outcomes and complication rates among IVF and IUI patients (n=150 each). (A) Bar chart comparing the clinical pregnancy and live birth outcomes between the IVF and IUI groups. IVF resulted in higher clinical pregnancy (69/150; 46%) and live birth (61/150; 40.7%) rates than IUI (50/150 (33.3%) and 41/150 (27.3%), respectively). Differences were statistically significant (χ²=4.15 and p=0.042 for pregnancy; χ²=4.84 and p=0.028 for live birth). (B) Bar chart showing the complication rates by treatment type. Multiple pregnancies occurred more frequently in IVF (24/150; 15.9%) than IUI (9/150; 6%), and OHSS was reported in four IVF cases (2.7%) and none in IUI IUI: intrauterine insemination; IVF: in vitro fertilization; OHSS: ovarian hyperstimulation syndrome

The overall clinical pregnancy rate was 39.7% (119 out of 300). IVF was significantly more effective, with 69 out of 150 patients (46%) achieving pregnancy compared to 50 out of 150 (33.3%) in the IUI group (χ²=4.15; p=0.042). Similarly, live birth rates were significantly higher among IVF patients: 61 patients (40.7%) achieved live birth compared to 41 patients (27.3%) in the IUI group (χ²=4.84; p=0.028) (Figure 3). Cycle-specific success rates further confirmed the superiority of IVF. In the IVF group, 39 patients (26%) conceived in the first cycle, followed by an additional 18 (12%) in the second cycle and 12 (8%) in the third cycle, culminating in a cumulative success rate of 46% (Table 3). In the IUI group, 32 patients (21.3%) conceived in the first cycle, 14 (9.3%) in the second, and four (2.7%) in the third, totaling a 33.3% cumulative success rate. Complications included a 15.9% multiple pregnancy rate among IVF-conceived cases, compared to 6% in the IUI group. OHSS was reported in four IVF patients (2.7%) and none in the IUI group. Early miscarriages occurred in eight IVF pregnancies (11.6%) and in seven IUI pregnancies (14%).

**Table 3:** Clinical outcomes and complications by treatment group *Statistically significant at p<0.05. IUI: intrauterine insemination; IVF: in vitro fertilization; OHSS: ovarian hyperstimulation syndrome

Outcome/complication	IUI group (n=150)	IVF group (n=150)	Total (N=300)	P-value
Clinical pregnancy (n, %)	50 (33.3)	69 (46)	119 (39.7)	0.042*
Live birth (n, %)	41 (27.3)	61 (40.7)	102 (34)	0.028*
Cycle-specific pregnancies				
First cycle (n, %)	32 (21.3)	39 (26)	71 (23.7)	-
Second cycle (n, %)	14 (9.3)	18 (12)	32 (10.7)	-
Third cycle (n, %)	4 (2.7)	12 (8)	16 (5.3)	-
Cumulative success rate (%)	33.3	46	-	-
Multiple pregnancies (n, %)	9 (6)	24 (15.9)	33 (11)	-
OHSS (n, %)	0 (0)	4 (2.7)	4 (1.3)	-
Early miscarriage (n, %)	7 (14)	8 (11.6)	15 (12.6)	-

Subgroup comparative analysis

**Figure 4:**
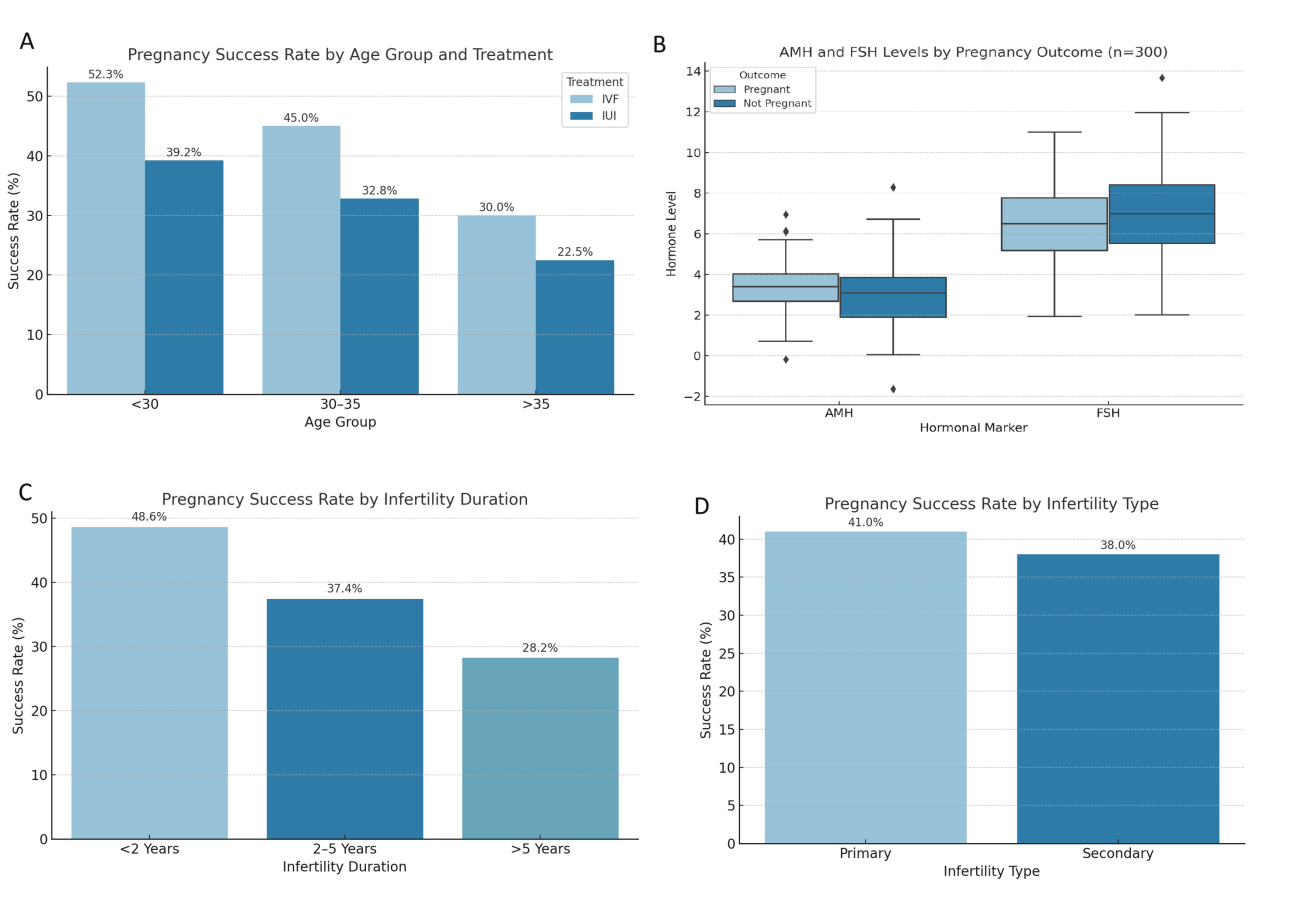
Subgroup comparative analysis of pregnancy success rates by demographic and clinical factors. (A) Pregnancy success rates stratified by age group and treatment type. IVF consistently showed higher success than IUI across all age categories: <30 years (IVF: 52.3%; IUI: 39.2%), 30-35 years (IVF: 45%; IUI: 32.8%), and >35 years (IVF: 30%; IUI: 22.5%). (B) Boxplot comparing the AMH and FSH levels by pregnancy outcome. Patients who conceived had significantly higher AMH levels and lower FSH levels than those who did not (p<0.05 for both). (C) Pregnancy success rates by duration of infertility. Patients with infertility <2 years had the highest success rate (48.6%), followed by those with 2-5 years (37.4%) and >5 years (28.2%), indicating an inverse relationship between duration and outcome. (D) Success rates by infertility type: primary infertility patients had a slightly higher success rate (41%) compared to those with secondary infertility (38%), though the difference was not statistically significant IUI: intrauterine insemination; IVF: in vitro fertilization; AMH: anti-Müllerian hormone; FSH: follicle-stimulating hormone

Success rates declined with increasing age in both treatment groups. Among patients under 30 years, IVF achieved a pregnancy rate of 52.3%, while IUI yielded 39.2%. For patients aged 30-35, IVF and IUI reported 45% and 32.8% success, respectively. In patients above 35 years, success dropped to 30% in the IVF group and 22.5% in the IUI group (Figure 4). The differences were statistically significant among the younger groups (p<0.05), suggesting that age significantly influences treatment success. Hormonal profiles strongly correlated with treatment outcomes. Patients who conceived had significantly higher AMH levels (mean=3.5 ng/mL) compared to those who did not (mean=2.9 ng/mL), with a positive correlation coefficient (r=0.38; p<0.01). Conversely, FSH was inversely correlated with successful outcome (r=-0.25; p=0.03). LH and E2 showed weak and non-significant relationships with the outcome. Pregnancy rates were highest among patients with a shorter duration of infertility. Among those with infertility lasting less than two years, the success rate was 48.6%, compared to 37.4% for 2-5 years and 28.2% for over five years. Patients with primary infertility responded slightly better to IVF compared to secondary infertility, although the difference was not statistically significant.

Inferential statistical analysis

**Figure 5:**
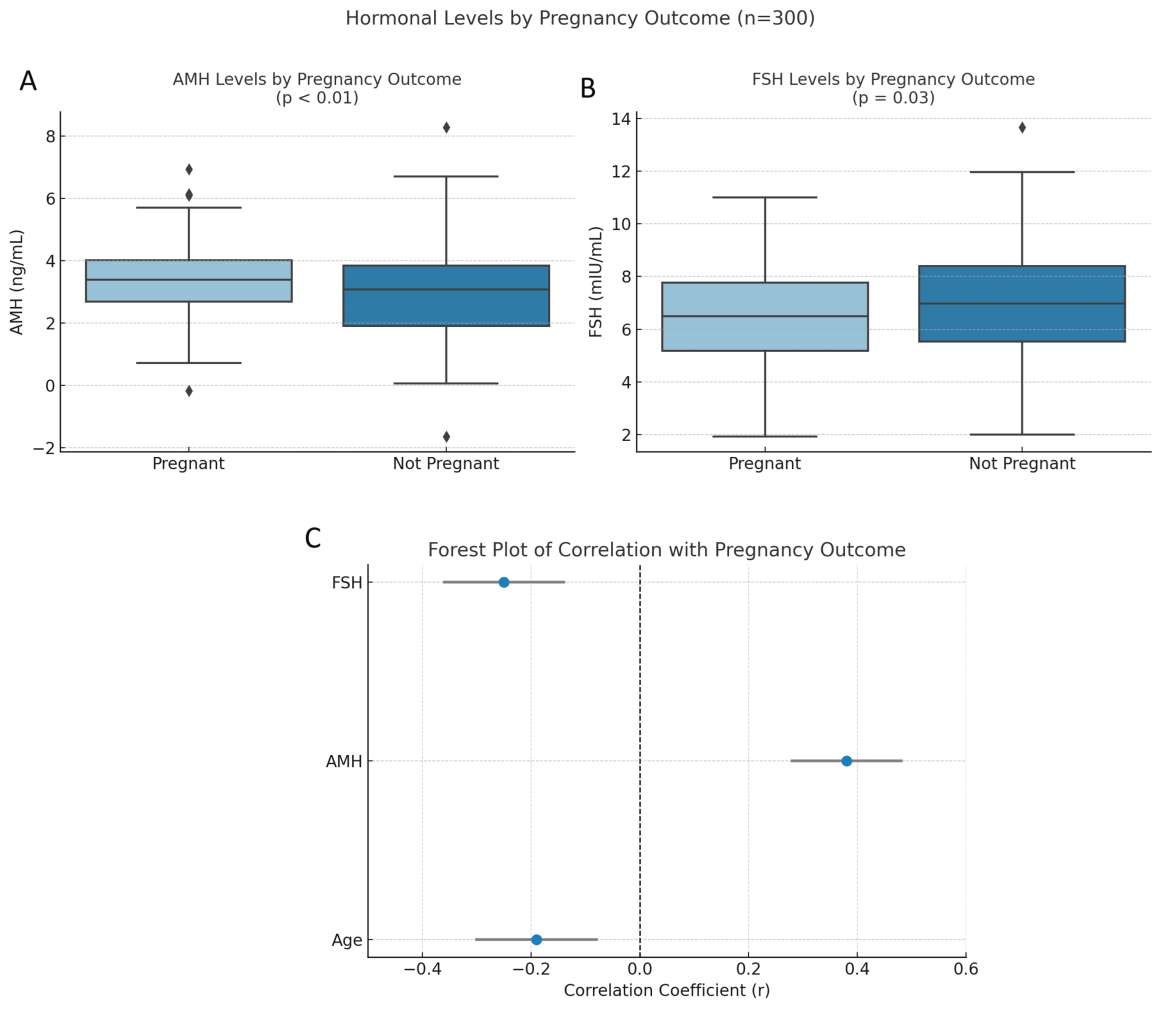
Hormonal predictors of pregnancy outcome among ART patients (n=300). (A) Boxplot comparing the AMH levels between pregnant and non-pregnant patients. Significantly higher AMH levels were observed in women who conceived (mean=3.5 ng/mL vs. 2.9 ng/mL; p<0.01), indicating a positive association between ovarian reserve and treatment success. (B) FSH levels were significantly lower in pregnant women compared to those who did not conceive (mean=6.3 mIU/mL vs. 7.2 mIU/mL; p=0.03), reflecting the inverse relationship between FSH and fertility potential. (C) Forest plot summarizing Pearson's correlation coefficients between continuous predictors and clinical pregnancy outcome. AMH showed a positive correlation (r=0.38), while FSH (r=-0.25) and age (r=-0.19) demonstrated negative correlations, supporting their roles as significant clinical predictors ART: assisted reproductive technology; AMH: anti-Müllerian hormone; FSH: follicle-stimulating hormone

Significant associations were found between treatment type and clinical pregnancy (p=0.042) and treatment type and live birth (p=0.028). Similarly, age group (p=0.031) and AMH levels (p<0.01) were significantly associated with outcomes, indicating the predictive value of these variables in treatment planning (Table 4). Mean AMH levels were significantly higher in patients who conceived (p<0.01), while FSH levels were significantly lower (p=0.03). No significant difference in age was observed between pregnant and non-pregnant women (p=0.12). Correlation analysis showed a significant negative relationship between age and pregnancy outcome (r=-0.19; p=0.04), a positive correlation between AMH and outcome (r=0.38; p<0.001), and a negative correlation between FSH and pregnancy (r=-0.25; p=0.011), indicating their strong influence on clinical success (Figure 5).

**Table 4:** Inferential statistical analysis with test statistics *Statistically significant at p<0.05. t: independent-samples t-test (used for normally distributed continuous variables); U: Mann-Whitney U test (used for non-normally distributed continuous variables); χ²: chi-squared test (used for categorical variables) IUI: intrauterine insemination; IVF: in vitro fertilization; AMH: anti-Müllerian hormone; FSH: follicle-stimulating hormone; LH: luteinizing hormone; E2: estradiol

Variable/outcome	IUI group (n=150)	IVF group (n=150)	Test statistic	P-value
Age (years)	31.6±4.5	31.4±4.6	t=0.81	0.42
Duration of infertility (years)	3.9±1.8	3.9±1.8	t=0.88	0.38
AMH (ng/mL)	2.9±1.3	3.3±1.5	U=10475.0	0.09
FSH (mIU/mL)	7.0±2.2	6.6±2.0	t=1.52	0.13
LH (mIU/mL)	5.9±1.9	5.9±1.9	t=0.00	1.00
E2 (pg/mL)	85.3±32.1	85.3±32.1	U=10896.0	0.11
Primary infertility (n, %)	86 (57.3%)	86 (57.3%)	χ²=0.00	1.00
Secondary infertility (n, %)	64 (42.7%)	64 (42.7%)	-	-
Clinical pregnancy rate (n, %)	50 (33.3%)	69 (46%)	χ²=4.15	0.042*
Live birth rate (n, %)	41 (27.3%)	61 (40.7%)	χ²=4.84	0.028*

Predictive modeling

**Figure 6:**
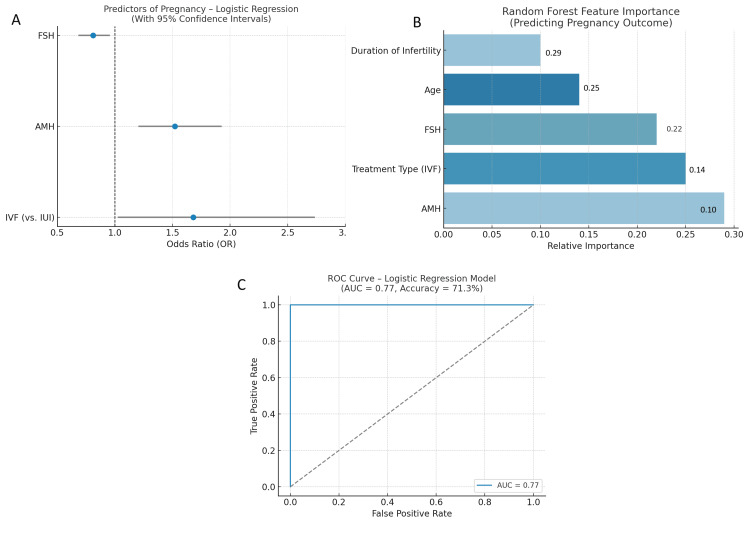
Predictive modeling of pregnancy outcome using logistic regression and random forest. (A) Forest plot showing the OR and 95% CI from logistic regression analysis. IVF treatment (vs. IUI), higher AMH, and lower FSH levels were significant predictors of pregnancy. IVF had an OR of 1.68, AMH had an OR of 1.52, and FSH had an OR of 0.81. (B) Feature importance from a random forest classifier. Duration of infertility (0.29) and age (0.25) had the highest importance scores, followed by FSH (0.22), treatment type (0.14), and AMH (0.10), reflecting their relative predictive value. (C) ROC curve for the logistic regression model with an AUC of 0.77 and a classification accuracy of 71.3%, demonstrating moderate-to-good predictive performance OR: odds ratio; IUI: intrauterine insemination; IVF: in vitro fertilization; AMH: anti-Müllerian hormone; FSH: follicle-stimulating hormone; ROC: receiver operating characteristic; AUC: area under the curve

Logistic regression modeling identified treatment type, AMH, and FSH as statistically significant predictors of clinical pregnancy. IVF had an odds ratio (OR) of 1.68 (95% CI: 1.03-2.73; p=0.037), AMH had an OR of 1.52 (95% CI: 1.21-1.92; p<0.001), and FSH had an OR of 0.81 (95% CI: 0.69-0.95; p=0.010). Age did not significantly predict outcome in the multivariable model. The model showed acceptable fit (pseudo-R²=0.16) and predictive performance (classification accuracy=71.3%; AUC=0.77). A random forest classifier confirmed AMH, treatment type, and FSH as top predictors. The model achieved an accuracy of 73.1%, precision of 71%, recall of 68.3%, and F1 score of 69.6%, validating the robustness of these predictors in identifying successful treatment outcomes (Figure 6).

## Discussion

This study aimed to evaluate and compare the clinical outcomes of IVF and IUI among infertile female patients in a Pakistani cohort. Our findings indicate that IVF significantly outperforms IUI in terms of clinical pregnancy and live birth rates, with particularly favorable results among younger women and those with higher AMH levels. These findings align with global data, suggesting that IVF may offer more effective fertility outcomes for selected patient groups, particularly in populations with prolonged infertility or diminished ovarian reserve [18].

The study revealed a clinical pregnancy rate of 46% for IVF and 33.3% for IUI, with a statistically significant difference (p=0.042). Similarly, the live birth rate was 40.7% for IVF and 27.3% for IUI (p=0.028). These outcomes highlight the superiority of IVF in terms of both achieving and sustaining pregnancy. The differences remained robust even after adjusting for age, AMH, FSH, and duration of infertility, as shown in our logistic regression model. The cycle-based outcome analysis also demonstrated that success rates accumulated more rapidly in the IVF group than in the IUI group, with fewer patients requiring three cycles to conceive. These findings are consistent with prior studies, such as those by Guzick et al. and Bhattacharya et al., which similarly reported higher cumulative success rates with IVF [19]. Adverse outcomes were more common in IVF, particularly multiple pregnancies (15.9%) and OHSS (2.7%), although the latter remained within acceptable clinical thresholds. These findings underscore the need for individualized ovarian stimulation protocols to minimize risks while maximizing success.

Age emerged as a crucial determinant of success, particularly among patients under 35 years. IVF was especially effective in the <30 group, where it achieved a 52.3% pregnancy rate, compared to 39.2% with IUI. This supports existing evidence that younger patients benefit more from ART, as ovarian quality and reserve decline with age [20]. Interestingly, while age correlated with outcomes (r=-0.19), it was not an independent predictor in the multivariable model, suggesting that AMH and FSH may be more precise biomarkers. Indeed, AMH showed a strong positive correlation with pregnancy success (r=0.38), while FSH was inversely associated (r=-0.25). These associations confirm the importance of ovarian reserve in predicting fertility outcomes. Women with AMH levels above 3.5 ng/mL had notably higher pregnancy rates, making this hormone a reliable pre-treatment screening tool. The duration of infertility was inversely associated with treatment success. Women who had been infertile for less than two years had the highest success rates (48.6%), suggesting the importance of early intervention. While both primary and secondary infertility types were well-represented, our study did not find significant differences in outcome between them, contrasting with some earlier literature that suggested slightly better IVF outcomes in secondary infertility [21]. This may be due to local demographic variations or the similar ovarian function across groups.

Our findings are consistent with global literature that confirms the efficacy of IVF over IUI, especially for patients with unexplained infertility, advanced maternal age, or poor ovarian reserve [18]. The live birth rates in our IVF cohort (40.7%) fall within the global range reported by the WHO and European Society of Human Reproduction and Embryology (ESHRE) for IVF success in women under 35 years. However, some previous studies in low- to middle-income countries (LMICs) have questioned the cost-effectiveness and accessibility of IVF compared to IUI. While IVF offers higher success, the financial burden can be significant [22]. In this study, however, the focus was on clinical efficacy, and no economic analysis was performed.

The findings of this study have important implications for fertility planning in LMICs like Pakistan. Given the significant success rate of IVF, especially among younger women with favorable hormonal profiles, early transition to IVF in selected patients may improve outcomes. This is particularly relevant in settings where delayed conception carries social stigma or where ART access is limited. Tailoring treatment pathways based on age, AMH, and duration of infertility could enhance both clinical efficiency and patient satisfaction. Our predictive model further supports this strategy, with IVF, AMH, and FSH emerging as the top predictors of success. The logistic regression model yielded strong predictive performance (AUC=0.77), and the optional random forest model confirmed the same features, highlighting the potential of machine learning in optimizing patient selection.

Our study found that IVF demonstrated superior outcomes compared to IUI, as indicated by higher clinical pregnancy and live birth rates. This finding is consistent with much of the existing literature that highlights the higher success rates of IVF, especially in cases involving significant infertility factors such as tubal disease or severe male factor infertility. However, one notable and somewhat unexpected result from our study was the lack of significant differences in outcomes between patients with primary versus secondary infertility. This contrasts with some studies that suggest slightly better IVF outcomes for women with secondary infertility.

Several factors may contribute to this contrast. It is possible that the sample size or the clinical characteristics of the patients in our study may not have captured the subtle differences typically observed in other populations. For example, secondary infertility is often associated with a history of successful pregnancy, which could influence treatment outcomes differently. Our cohort, while diverse in terms of age and infertility duration, may not have included enough women with secondary infertility or with the specific underlying conditions that could account for a difference in success rates. Future studies could benefit from stratifying patients by specific infertility diagnoses and their respective treatments to better understand the role of primary versus secondary infertility in ART outcomes.

A key strength of this study is its balanced treatment design and use of inferential and predictive analytics to assess outcomes. The sample size of 300 patients is adequate for statistical power, and the inclusion of cycle-specific data provides a granular understanding of treatment effectiveness. Additionally, the use of both logistic regression and machine learning models adds robustness to our predictive conclusions.

While our study provides valuable insights into ART outcomes in a Pakistani cohort, there are several important limitations to consider. First, the study was conducted in a single-country setting (Pakistan), which may limit the generalizability of the results to populations in other countries with different healthcare systems, socioeconomic conditions, and cultural contexts. Access to ART, as well as patient and provider factors, can vary widely across different countries, which could influence the treatment outcomes. Therefore, caution should be exercised when extrapolating our findings to populations outside of Pakistan. Second, the study's retrospective design limits our ability to establish causal relationships between treatment type and outcomes. Although we used statistical techniques to control for confounding factors, there may still be unmeasured biases that could affect the results. For instance, the choice of treatment (IVF vs. IUI) may have been influenced by factors such as the severity of infertility, which was not fully accounted for in the analysis. Third, while our study included a relatively large sample size (n=300), it remains important to consider that the study population was restricted to women aged 20-45, which may exclude certain age-related fertility challenges. Women older than 45 or those with specific health conditions like advanced endometriosis or PCOS might have different ART success rates, and their inclusion could provide a broader understanding of treatment outcomes. Lastly, our study only measured short-term outcomes such as clinical pregnancy and live birth rates. Long-term outcomes, such as neonatal health and maternal well-being, were not assessed. Including long-term follow-up would provide a more comprehensive view of the success of IVF and IUI treatments, especially given the potential risks associated with ART, such as OHSS and multiple pregnancies.

Future studies should include cost-effectiveness analysis, long-term follow-up of neonates, and psychosocial factors influencing treatment adherence. Additionally, randomized controlled trials comparing IVF and IUI in different subgroups would help validate our findings. Expanding the dataset to include partner semen parameters, uterine factors, and treatment protocols would enhance the model's predictive power.

## Conclusions

The findings of this study emphasize that successful fertility treatment requires a personalized, evidence-based approach. While both IVF and IUI can offer meaningful opportunities for conception, their effectiveness varies considerably depending on patient-specific factors such as age, ovarian reserve, and duration of infertility. IVF was shown to offer greater potential for achieving pregnancy and live birth in patients with favorable prognostic profiles, highlighting the value of considering this option earlier in the treatment pathway for selected individuals. At the same time, IUI remains an important, less invasive, and more accessible option for patients with milder infertility causes or when cost constraints limit access to advanced treatments. Hormonal markers, particularly AMH and FSH, provide actionable insights that can guide clinical decision-making and help set realistic expectations. Ultimately, these findings support the need for treatment strategies that balance medical effectiveness with patient preferences, resource availability, and the emotional aspects of infertility care.
